# Identification of Quorum-Sensing Signal Molecules and a Biosynthetic Gene in *Alicycliphilus* sp. Isolated from Activated Sludge

**DOI:** 10.3390/s16081218

**Published:** 2016-08-02

**Authors:** Tomohiro Morohoshi, Noriya Okutsu, Xiaonan Xie, Tsukasa Ikeda

**Affiliations:** 1 Department of Material and Environmental Chemistry, Graduate School of Engineering, Utsunomiya University, 7-1-2 Yoto, Utsunomiya, Tochigi 321-8585, Japan; noriya.okutsu@kurita.co.jp; 2 Center for Bioscience Research and Education, Utsunomiya University, 350 Mine-machi, Utsunomiya, Tochigi 321-8505, Japan; xie@cc.utsunomiya-u.ac.jp

**Keywords:** quorum sensing, *N*-acylhomoserine lactone, activated sludge, *Alicycliphilus* sp.

## Abstract

Activated sludge is a complicated mixture of various microorganisms that is used to treat sewage and industrial wastewater. Many bacteria produce *N*-acylhomoserine lactone (AHL) as a quorum-sensing signal molecule to regulate the expression of the exoenzymes used for wastewater treatment. Here, we isolated an AHL-producing bacteria from an activated sludge sample collected from an electronic component factory, which we named *Alicycliphilus* sp. B1. Clone library analysis revealed that *Alicycliphilus* was a subdominant genus in this sample. When we screened the activated sludge sample for AHL-producing strains, 12 of 14 the AHL-producing isolates were assigned to the genus *Alicycliphilus*. A putative AHL-synthase gene, ALISP_0667, was cloned from the genome of B1 and transformed into *Escherichia coli* DH5α. The AHLs were extracted from the culture supernatants of the B1 strain and *E. coli* DH5α cells harboring the ALISP_0667 gene and were identified by liquid chromatography-mass spectrometry as *N*-(3-hydroxydecanoyl)-l-homoserine lactone and *N*-(3-hydroxydodecanoyl)-l-homoserine lactone. The results of comparative genomic analysis suggested that the quorum-sensing genes in the B1 strain might have been acquired by horizontal gene transfer within activated sludge.

## 1. Introduction

Activated sludge systems are used for the treatment of wastewater produced by various industries [1] and are complicated mixtures of many types of microorganisms. One type, aerobic bacteria, contributes to the decomposition of organic material. Management of activated sludge systems is mainly based on empirical techniques. However, unexpected problems due to bacterial behaviors sometimes occur as a result of conditional changes in parameters such as temperature, flow rate, and sewage composition. Therefore, for stable operation of these systems, it is necessary to understand the bacterial interactions in activated sludge. Quorum sensing is a cell density-dependent cell-to-cell communication system that regulates gene expression [2]. In gram-negative bacteria, *N*-acylhomoserine lactones (AHLs) are used as the quorum-sensing signaling molecules [3]. When the concentration of AHL accumulates and reaches a threshold, LuxR family proteins, which function as AHL receptors, form complexes with AHL and activate the expression of target genes involved in bioluminescence, antibiotic production, biofilm formation, etc. [4]. Various AHL-producing bacteria have been isolated from activated sludge [5]. These bacteria regulate the expression of exoenzymes, which are involved in wastewater treatment, through AHL-mediated quorum-sensing systems [1,5]. In the microbial communities in aerobic sludge granules, AHL-mediated quorum-sensing contributes to the initiation of granulation and the biosynthesis of extracellular polymeric substances [6]. These results suggest that quorum sensing has a positive effect on wastewater treatment. However, quorum sensing has also been associated with the formation of membrane biofouling in a membrane bioreactor system [7]. In this case, inhibition of quorum sensing was an effective strategy to control membrane biofouling [7].

The semiconductor and electronic parts manufacturing industries generate high-strength wastewater containing dimethyl sulfoxide, tetramethylammonium, isopropyl alcohol, and acetone [8]. However, the microbial communities in the activated sludge used for electronic wastewater treatment have not yet been elucidated. In a previous study, we isolated *Alicycliphilus* sp. B1 as an AHL-producing strain from an activated sludge sample collected from an electronic component factory [9]. The genus *Alicycliphilus* is in the family Comamonadaceae in the β-subclass of Proteobacteria [10]. AHL-mediated quorum sensing by *Alicycliphilus* sp. B1 might affect its ability to degrade high-strength substances. Two strains of the genus *Alicycliphilus*, *A. denitrificans* K601 and BC, were previously isolated from a municipal sewage treatment plant through cyclohexanol and benzene-degrading chlorate-reducing enrichment, respectively [11]. However, these strains have not been reported to be AHL-producing bacteria. In this study, we investigated the distribution of AHL-producing *Alicycliphilus* strains in activated sludge from a wastewater treatment facility for an electronic component factory, and characterized an AHL biosynthetic gene in *Alicycliphilus*.

## 2. Materials and Methods

### 2.1. Clone Library Analysis

An activated sludge sample was obtained from a wastewater treatment plant at an electronic component factory. Total DNA was extracted with Cica Geneus DNA Extraction Reagent (Kanto Kagaku, Singapore). The 16S rRNA genes were amplified by PCR with Blend Taq plus DNA polymerase (Toyobo) and universal primers 27f (5’-AGA GTT TGA TCM TGG CTC AG-3’) and 1525r (5’-AAG GAG GTG WTC CAR CC-3’). The PCR was performed using the following cycling parameters: 94 °C for 30 s, 55 °C for 30 s, and 72 °C for 1 min for 30 cycles. The PCR products were cloned into a pGEM-T easy cloning vector (Promega, Madison, WI, USA). The 16S rRNA gene-encoding regions were sequenced using BigDye Terminator ver. 3.1 and a 3730xl DNA Analyzer (Applied Biosystems, Foster City, CA, USA).

### 2.2. Screening and Identification of AHL-Producing Bacteria

The activated sludge sample was serially diluted in sterilized distilled water and spread on R2A agar medium plates (Becton, Dickinson and Company, Franklin Lakes, NJ, USA), and the plates were incubated at 30 °C for 72 h. Colonies were randomly chosen and inoculated onto fresh R2A agar medium. AHL production by isolates was detected by two AHL biosensors, *Chromobacterium violaceum* CV026 [12] and VIR07 [13]. The two AHL reporter strains were cross-streaked onto the side of isolates. After incubation at 30 °C for 24 h, bacteria that induced the production of purple pigment in the AHL reporter strains were selected as AHL-producing bacteria. The 16S rRNA gene-encoding regions of these strains were amplified and sequenced by the method described above. The closest type strain to each 16S rRNA gene sequence was determined using the RDP II sequence match tool [14].

### 2.3. Cloning of the AHL-Synthase Gene from Alicycliphilus sp. B1

The putative AHL-synthase gene (ALISP_0667) and the surrounding sequence were amplified by KOD FX Neo DNA polymerase (Toyobo) using the following primers, (5’-CCG TGC AAT CGC AGC AAA ATC GGT AGT TGC GTT GC-3’) and (5’-GAA CAT CGC CAG CAG CAT TTC GGC GAG ACG GCA TC-3’). The PCR was performed with the following cycling parameters: 94 °C for 30 s and 68 °C for 2 min for 30 cycles. Overhanging deoxyadenosines were added to the 3’-ends of the PCR product by Blend Taq plus DNA polymerase, and the resulting product was cloned into the pGEM-T easy cloning vector. The constructed plasmid, pGEM-B1luxI, was transformed into *Escherichia coli* DH5α.

### 2.4. Extraction and Identification of AHLs

*Alicycliphilus* sp. B1 and *E. coli* DH5α cells harboring pGEM-B1luxI were inoculated into 4 mL of R2A medium and LB medium containing 100 µg/mL ampicillin, respectively. After cultivation at 30 °C for 18 h with shaking, 2 mL of the cultures were transferred into 200 mL of fresh medium and incubated at 30 °C for 24 h with shaking. Cells were pelleted by centrifugation at 10,000× *g* for 5 min. The culture supernatant was concentrated by evaporation at 40 °C using a rotary evaporator. The concentrated supernatant was extracted with 3 volumes of ethyl acetate in a separatory funnel. The extract was evaporated to dryness using a rotary evaporator and was then dissolved in 500 μL of dimethylsulfoxide. Full-grown CV026 or VIR07 cultures were mixed with LB agar and plated. Then, 8-mm paper disks, to which putative AHL samples were applied, were placed on the LB agar plates containing CV026 or VIR07 cells. After overnight incubation at 30 °C, the presence of AHL was assessed as the appearance of purple pigment. The chemical structures of the extracted AHLs were analyzed by liquid chromatography-mass spectrometry (LC-MS) as described previously [15]. Briefly, AHL samples (2 μL) were injected into the LC-MS system. Q1 was set to scan a mass range of *m*/*z* 80 to 500 Da. Q3 was monitored for the product ion that indicates the presence of a lactone ring at *m*/*z* 102. AHLs were analyzed by LC-MS/MS with MS/MS and precursor ion scanning experiments and identified by comparison to synthetic AHLs standards.

### 2.5. Chemical Synthesis of AHL Standards

*N*-(3-oxodecanoyl)-l-homoserine lactone (3-oxo-C10-HSL) and *N*-(3-oxododecanoyl)-l-homoserine lactone (3-oxo-C12-HSL) were synthesized by a previously described method [16]. *N*-(3-hydroxydecanoyl)-l-homoserine lactone (3-OH-C10-HSL) and *N*-(3-hydroxydodecanoyl)-l-homoserine lactone (3-OH-C12-HSL) were prepared from 3-oxo-C10-HSL and 3-oxo-C12-HSL via reduction of the ketone with NaBH_4_ using a previously described method [17].

## 3. Results and Discussion

### 3.1. Phylogenetic Analysis of the Clone Libraries from Activated Sludge

To determine the composition of the bacterial community in the activated sludge from the wastewater treatment plant of an electronic component factory, clone libraries of the 16S rRNA genes were prepared. A total of 95 clones were sequenced and analyzed phylogenetically. The results showed that *Runella* was the predominant genus in the activated sludge sample (72.6%; Table 1), followed by *Methylibium*, *Acidovorax*, and *Alicycliphilus* (8.4%, 5.3%, and 3.2%, respectively).

### 3.2. Isolation and Identification of AHL-Producing Bacteria from Activated Sludge

A total of 48 bacterial strains were isolated from the activated sludge sample. AHL-producing bacteria were screened by using the AHL-reporter strains *C. violaceum* CV026 and VIR07. Of the 48 bacterial strains, 14 strains showed AHL-producing activity. The 16S rRNA genes of the AHL-producing isolates were amplified by PCR and sequenced for identification (Table 2). Twelve of the AHL-producing strains showed high similarity (>99%) to *Alicycliphilus denitrificans* K601^T^. However, we did not isolate AHL-producing strains belonging to the other predominant or subdominant genera, such as *Runella*, *Methylibium*, and *Acidovorax*. Two AHL-producing strains showed high similarity (98.3%) to *Pseudomonas azotoformans* IAM1603^T^. However, the clone library analysis demonstrated that the genus *Pseudomonas* was a minor member in this sample (Table 1). These results demonstrated that strains in the genus *Alicycliphilus* were the most common AHL-producing strains in this activated sludge sample.

### 3.3. Analytical Identification of AHL Molecules

**Figure 1:**
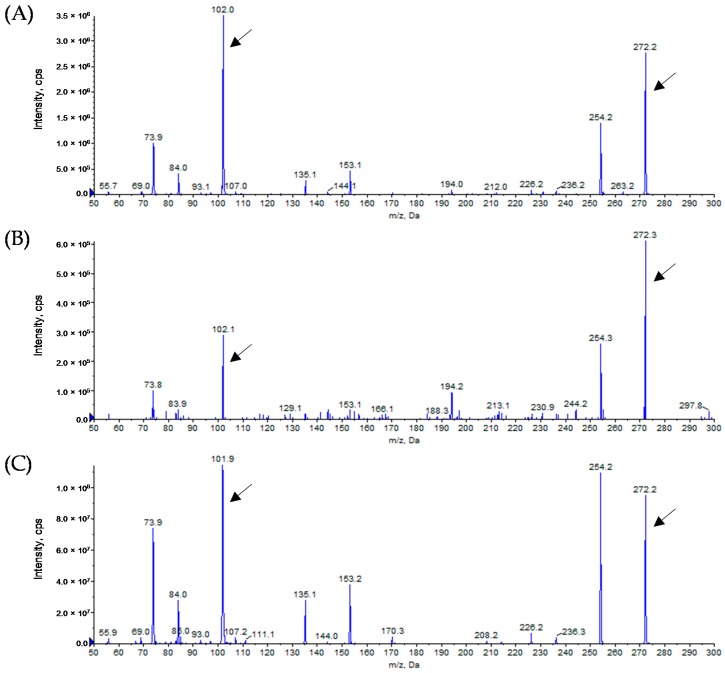
Mass spectra of the AHLs extracted from the cell-free supernatants of *Alicycliphilus* sp. B1 (**A**) and *E. coli* DH5α harboring pGEM-B1luxI (**B**) and a *N*-(3-oxodecanoyl)-l-homoserine lactone (3-OH-C10-HSL) standard (**C**). After fractionation of the cell-free extracts by reverse-phase HPLC, the (ESI-MS/MS fragment peaks of the AHLs were analyzed. All peaks of 3-OH-C10-HSL (*m/z* 272) along with the product ion peaks (*m/z* 102) are marked with arrows.

**Figure 2:**
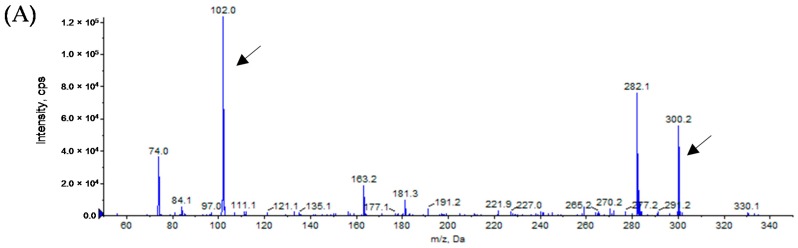

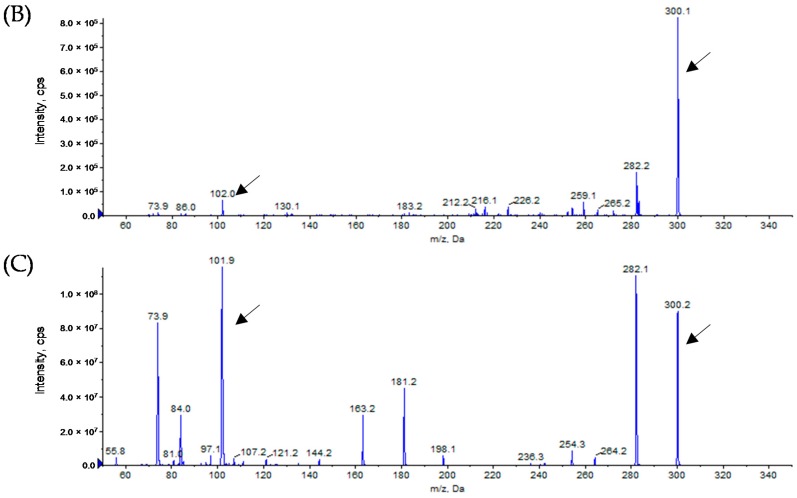
Mass spectra of the AHLs extracted from the cell-free supernatants of *Alicycliphilus* sp. B1 (**A**) and *E. coli* DH5α harboring pGEM-B1luxI (**B**) and a 3-OH-C12-HSL standard (**C**). After fractionation of the cell-free extracts by reverse-phase HPLC, the ESI-MS/MS fragment peaks of the AHLs were analyzed. All peaks of 3-OH-C12-HSL (*m/z* 300) along with the product ion peaks (*m/z* 102) are marked with arrows.

To determine the structure of the AHLs produced by *Alicycliphilus* sp. B1, a sample was extracted from the supernatant of a B1 culture. The presence of AHLs was assessed by using LB agar plates containing *C. violaceum* CV026 and VIR07. AHL extracts from B1 slightly stimulated violacein production in VIR07 cells, but not in CV026 cells. HPLC fractionation followed by MS analysis of the AHL extracts was performed to identify the AHLs. In the AHL extracts from B1, we identified 3-OH-C10-HSL and 3-OH-C12-HSL (Figure 1 and Figure 2), suggesting that *Alicycliphilus* sp. B1 produces these two types of AHLs. In a previous study, we identified a putative AHL-synthase gene homolog (ALISP_0667) in contig 11 of the draft genome sequence of B1 [9]. To determine whether expression of the ALISP_0667 gene results in AHL production, an ALISP_0667-encoding plasmid, pGEM-B1luxI, was prepared and transformed into *E. coli* DH5α. An AHL sample was extracted from the supernatant of a culture of *E. coli* DH5α harboring pGEM-B1luxI. The LC-MS analysis showed 3-OH-C10-HSL and 3-OH-C12-HSL in the extract from *E. coli* DH5α harboring pGEM-B1luxI (Figure 1 and Figure 2). The AHL profile of the extracts from *E. coli* DH5α harboring pGEM-B1luxI matched that of *Alicycliphilus* sp. B1. In addition, no other AHL-synthase gene homologues were found in the genome sequence of B1 [9]. These results demonstrated that only the product of ALISP_0667 is required for of the synthesis of these two AHL molecules.

### 3.4. Comparative Genomic Analysis of Alicycliphilus Strains

**Figure 3:**
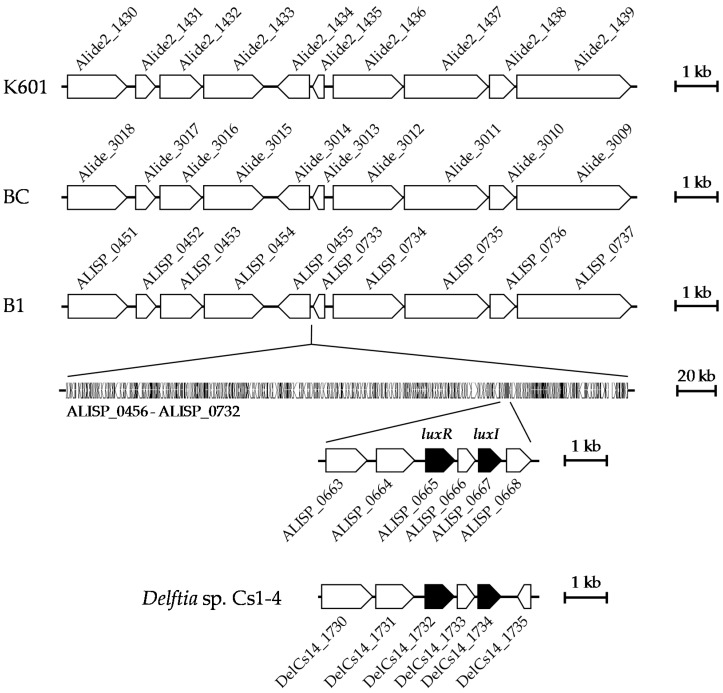
The chromosomal locus in *Alicycliphilus* sp. B1 containing the AHL synthase gene. Size, position, and orientation of the coding sequences in the genomes of *A. denitrificans* K601 and BC, *Alicycliphilus* sp. B1, and *Delftia* sp. Cs1-4 genes are shown as pentagons. Quorum sensing-related genes are shown as filled pentagons. The scale bars display the genome length (1 or 20 kbp).

In a previous study, the complete genome sequences of *A. denitrificans* BC and K601 were reported [18]. However, a BLAST search failed to identify any AHL synthase genes in these genome sequences. We compared the nucleotide sequence of a large contig containing ALISP_0667 to the genome sequences of *A. denitrificans* BC and K601 and found that an approximately 291-kbp region, including ALISP_0456 through ALISP_0732, was only found in the draft genome of *A. denitrificans* B1 (Figure 3). The nucleotide sequences of the upstream (ALISP_0451 to ALISP_0455) and downstream (ALISP_0733 to ALISP_0737) regions were highly conserved among the genome sequences of *A. denitrificans* BC and K601 (Figure 3). The inserted DNA sequence (ALISP_0456 to ALISP_0732) showed some similarity (52.0% identity with gaps) to a genome region in *Delftia* sp. Cs1-4 (DelCs14_1619–DelCs14_1846). In particular, the amino acid sequences of the AHL synthase gene (ALISP_0667) and AHL receptor gene (ALISP_0665) from *Alicycliphilus* sp. B1 showed high sequence identities with DelCs14_1734 (76.0%) and DelCs14_1732 (76.4%) from *Delftia* sp. Cs1-4, respectively (Figure 3). Although some *Delftia* strains have the ability to degrade AHL [19,20], there are no reports on the production of AHL by *Delftia* species. Bacterial strains belonging to genus *Delftia* are sometimes isolated from activated sludge and have the ability to degrade aromatic compounds [21,22]. Thus, there is a possibility that the quorum-sensing genes in *Alicycliphilus* sp. B1 were acquired from the genome of *Delftia* sp. in activated sludge by horizontal gene transfer.

## 4. Conclusions

In summary, we demonstrated for the first time the structure of the AHLs produced by an *Alicycliphilus* sp. strain isolated from an activated sludge sample at an electronic component factory. It has been reported that bacteria in the genus *Alicycliphilus* have the ability to degrade various high-strength chemical compounds, such as cyclohexanol, benzene, and acetone [11,23]. The wastewater from electronic component factories frequently contains high-strength compounds such as dimethyl sulfoxide, tetramethylammonium, and isopropyl alcohol. Since activated sludge has a high cell density, it is possible that the quorum-sensing systems in *Alicycliphilus* are associated with the degradation of the high-strength compounds. However, the relationship between AHL-mediated quorum sensing in *Alicycliphilus* sp. B1 and wastewater treatment activity has not yet been elucidated. Future studies of AHL-mediated quorum sensing in *Alicycliphilus* might be useful for improving the treatment of wastewater from electronic component factories.

## Figures and Tables

**Table 1:** Taxonomic variation in the bacterial communities of the activated sludge sample.

Closest Genera	Clones	%
*Runella*	69	72.6
*Methylibium*	8	8.4
*Acidovorax*	5	5.3
*Alicycliphilus*	3	3.2
*Leptothrix*	2	2.1
*Methyloversatilis*	2	2.1
Others	6	6.3
Total	95	100

**Table 2:** Identification and characterization of *N*-acylhomoserine lactone (AHL)-producing strains. AHL-induced violacein production by CV026 or VIR07 is represented as plus and minus signs (+, low induction; ++, high induction; -, no induction).

Strains	Related Type Strain	CV026	VIR07
B1	*Alicycliphilus denitrificans* K601^T^	-	++
B3	*Alicycliphilus denitrificans* K601^T^	-	++
B9	*Alicycliphilus denitrificans* K601^T^	-	++
B12	*Alicycliphilus denitrificans* K601^T^	-	++
C3	*Alicycliphilus denitrificans* K601^T^	-	++
C4	*Alicycliphilus denitrificans* K601^T^	-	++
C5	*Alicycliphilus denitrificans* K601^T^	-	++
C10	*Alicycliphilus denitrificans* K601^T^	-	++
C11	*Alicycliphilus denitrificans* K601^T^	-	++
D1	*Pseudomonas azotoformans* IAM1603^T^	-	+
D2	*Pseudomonas azotoformans* IAM1603^T^	-	+
D3	*Alicycliphilus denitrificans* K601^T^	-	++
D10	*Alicycliphilus denitrificans* K601^T^	-	++
D11	*Alicycliphilus denitrificans* K601^T^	-	++
